# Rab Interacting Molecules 2 and 3 Directly Interact with the Pore-Forming Ca_V_1.3 Ca^2+^ Channel Subunit and Promote Its Membrane Expression

**DOI:** 10.3389/fncel.2017.00160

**Published:** 2017-06-08

**Authors:** Maria M. Picher, Ana-Maria Oprişoreanu, SangYong Jung, Katrin Michel, Susanne Schoch, Tobias Moser

**Affiliations:** ^1^Institute for Auditory Neuroscience and InnerEarLab, University Medical Center GöttingenGöttingen, Germany; ^2^Synaptic Nanophysiology Group, Max Planck Institute for Biophysical ChemistryGöttingen, Germany; ^3^Göttingen Graduate School for Neurosciences and Molecular Biosciences, University of GöttingenGöttingen, Germany; ^4^Institute of Neuropathology and Department of Epileptology, University of BonnBonn, Germany; ^5^Neuro Modulation and Neuro Circuitry Group, Singapore Bioimaging Consortium (SBIC), Biomedical Sciences InstitutesSingapore, Singapore; ^6^Collaborative Research Center 889, University of GöttingenGöttingen, Germany

**Keywords:** active zone, ribbon synapse, hair cell, channel clustering, exocytosis, hearing

## Abstract

Rab interacting molecules (RIMs) are multi-domain proteins that positively regulate the number of Ca^2+^ channels at the presynaptic active zone (AZ). Several molecular mechanisms have been demonstrated for RIM-binding to components of the presynaptic Ca^2+^ channel complex, the key signaling element at the AZ. Here, we report an interaction of the C_2_B domain of RIM2α and RIM3γ with the C-terminus of the pore-forming α–subunit of Ca_V_1.3 channels (Ca_V_1.3α1), which mediate stimulus-secretion coupling at the ribbon synapses of cochlear inner hair cells (IHCs). Co-expressing full-length RIM2α with a Ca^2+^ channel complex closely resembling that of IHCs (Ca_V_1.3α1-Ca_V_ß2a) in HEK293 cells doubled the Ca^2+^-current and shifted the voltage-dependence of Ca^2+^ channel activation by approximately +3 mV. Co-expression of the short RIM isoform RIM3γ increased the Ca_V_1.3α1-Ca_V_ß2a-mediated Ca^2+^-influx in HEK293 cells, but disruption of RIM3γ in mice left Ca^2+^-influx in IHCs and hearing intact. In conclusion, we propose that RIM2α and RIM3γ directly interact with the C-terminus of the pore-forming subunit of Ca_V_1.3 Ca^2+^ channels and positively regulate their plasma membrane expression in HEK293 cells.

## Introduction

Ca^2+^-influx through voltage-gated Ca^2+^ channels triggers the fusion of synaptic vesicles at the presynaptic active zone (AZ). The molecular mechanisms regulating the number and function of presynaptic Ca^2+^ channels are only partially understood but thought to involve presynaptic multidomain proteins such as Rab3 interacting molecule (RIM; Coppola et al., 2001; Kiyonaka et al., 2007; Han et al., 2011; Kaeser et al., 2011; Jung et al., 2015), RIM-binding protein (Liu et al., 2011; Acuna et al., 2015; Li and Kavalali, 2015; Müller et al., 2015) and Bassoon (Frank et al., 2010; Davydova et al., 2014). Four genes (RIMS1–4) encode the seven members of the RIM protein family (RIM1α, β; RIM2 α, β, γ; RIM3γ and RIM4γ), all exhibiting a C-terminal C_2_B domain, while their complement of further domains differs. The long RIM isoforms (RIM1α, β; RIM2α, β) contain an additional C_2_A domain, a PDZ domain, a zinc-finger domain and, for the α-isoforms, an N-terminal α-helix (Wang and Südhof, 2003). RIM1/2 interact with the pore-forming Ca_V_α1 subunit of Ca_V_2 channels through their central PDZ-domain (Ca_V_2.X (Kaeser et al., 2011)). Furthermore, they have been reported to bind via their C-terminal C_2_A and C_2_B domains to the auxiliary β (Ca_V_β) subunit (Kiyonaka et al., 2007; Gebhart et al., 2010; Gandini et al., 2011) as well as to the “synaptic protein interaction” motif (synprint motif; cytoplasmic linker between domains II and III) of the Ca_V_2.2α1 and Ca_V_1.2α1 subunits, which, however, was not found for the Ca_V_1.3α1 subunit (Coppola et al., 2001). In addition, RIMs are indirectly linked to Ca^2+^ channels by RIM-binding proteins (Hibino et al., 2002; Kaeser et al., 2011; Liu et al., 2011). A regulation of biophysical Ca^2+^ channel properties has been demonstrated in heterologous expression systems for RIM1 and RIM2 (Kiyonaka et al., 2007; Gebhart et al., 2010). The extent of this regulation depended on the respective Ca_V_ß subunit co-expressed and was least prominent for Ca_V_1.3 in the presence of palmitoylated Ca_V_ß2a (Gebhart et al., 2010; Gandini et al., 2011) that we postulate to be the predominant Ca_V_ß subunit in inner hair cells (IHCs; Neef et al., 2009).

Disruption of RIM1 and/or RIM2 was shown to reduce the number of Ca^2+^ channels at the presynaptic AZ of several synapses (Han et al., 2011, 2015; Kaeser et al., 2011; Kintscher et al., 2013; Jung et al., 2015). On top of a general reduction in the Ca^2+^-current upon disruption of RIM2α and RIM2ß in IHCs, a preferential loss of synaptic Ca^2+^ channels was reported based on comparing the reduction of the AZ Ca^2+^-signal and the whole-cell Ca^2+^-current (Jung et al., 2015). It is commonly assumed that RIM positively regulates the number of Ca^2+^ channels at the AZ by directly and indirectly interacting with the channel. However, it is less clear whether and how RIMs function in Ca^2+^ channel regulation intersects with similar roles of the auxiliary Ca^2+^ channel subunits Ca_V_ß and Ca_V_α2δ that have been described (Bichet et al., 2000; Neef et al., 2009; Altier et al., 2011; Dolphin, 2012; Hoppa et al., 2012; Fell et al., 2016; Wang et al., 2016). In IHCs, for example, Ca_V_ß 2 is critical for establishing sufficient membrane expression of Ca_V_1.3 (Neef et al., 2009) that mediates more than 90% of the IHC Ca^2+^-influx (Platzer et al., 2000; Brandt et al., 2003; Dou et al., 2004). However, despite the likely prevailing role of palmitoylated Ca_V_ß2a in IHCs that occludes effects of RIM2 on Ca_V_1.3 channels in heterologous expression systems (Gebhart et al., 2010), a dramatic loss of Ca^2+^ channels upon genetic disruption of RIM2 was observed in IHCs (Jung et al., 2015). Therefore, we reasoned that RIM2 might employ mechanisms beyond the Ca_V_ß interaction to promote the large complement of synaptic Ca^2+^ channels in IHCs. Specifically, we were interested to explore whether RIM2 could directly interact with the Ca_V_1.3α1 subunit. However, Ca_V_1.3α1 neither contains the C-terminal PDZ-binding motif for the interaction with RIM1/2 PDZ-domains (Kaeser et al., 2011; DDWC (Ca_V_2.1); DHWC (Ca_V_2.2); DDKC (Ca_V_2.3) vs. ITTL (Ca_V_1.3), which binds other PDZ domain proteins of IHCs such as harmonin (Gregory et al., 2011)) nor a synprint site, which binds C_2_-domains of RIM1/2 (Sheng et al., 1997; Chapman and Davis, 1998; Coppola et al., 2001) raising the question how RIM1/2 promotes Ca_V_1.3 channel abundance at IHC AZs. The same question applies to RIM3γ that was also found at IHC ribbon synapses (Jung et al., 2015) and the function of which at the presynaptic AZ has remained elusive. Here, we combined biochemical, physiological and morphological approaches to further investigate the interplay of RIMs and the Ca_V_1.3 channel complex.

## Materials and Methods

### Animals

Knock-out mice for RIM3γ were generated utilizing ES cells produced by the international Knockout Mouse Project (KOMP) consortium (Rims3tm1a(KOMP)Wtsi; ES cell line JM8A3.N1; targeting project CSD34392). The line obtained after germ line transmission constitutes a “knock-out first” allele, in which insertion of a splice acceptor-lacZ gene trap cassette disrupts the endogenous RIM3γ transcript resulting in a constitutive knock-out (*RIM3*γ^−/−^). ES cells were injected into Balb/c mice. The resulting chimeric mice were monitored by coat color and genotyped by PCR. The following primers were used for the *RIM3*γ^−/−^ line: RIM3γ 5′-GGACCACACTGCAATG-CTAA-3′ and 5′-CCCTTCAGTCTTCCTGTCCA-3′ product size 618 base pairs; *RIM3*^+/+^ 5′-GGACCACACTGCAATGCTAA-3′ and 5′-ACCAGACTCCAAAGCCCTC-3′ product size 324 base pairs. All analyses were carried out with littermates of heterozygous matings. In all animal experiments knock-out animals were compared to littermate controls, respectively. All experiments were performed in compliance with the national animal care guidelines and were approved by the board for animal welfare of the University Medical Center Göttingen, the University of Bonn and the animal welfare office of the state of Lower Saxony and North Rhine-Westphali.

### mRNA Isolation and cDNA Synthesis

Total mRNA was obtained from microdissected mouse brain tissue using Dynabeads mRNA DIRECT Micro Kit according to the manufacturer’s (Life Technologies) instructions. cDNA was synthesized from purified mRNA by reverse transcription using the RevertAidH Minus Strand cDNA Synthesis Kit (Fermentas) and compromised oligo dT primers according to the manufacture’s manual. cDNA samples were stored at −20°C. For quantitative real time PCR the Maxima Probe/Rox qPCR Master Mix (Thermo Fischer) together with Taqman gene expression assays (Applied Biosystem) was used according to the following protocol: experiments were performed in triplicates on an ABI Prism 9700HT system (PE Applied Biosystems, Foster City, CA, USA). Gene expression was analyzed as relative gene expression in comparison to the internal reference gene synaptophysin. Therefore gene expression was calculated as 2-∆ct (D cycle threshold value (ct) = ct of the analyzed gene − ct synaptophysin).

### Preparation of Protein Homogenates and Immunoblotting

Cell lysates from brain tissue were prepared from microdissected brain areas. Directly after preparation tissue samples were frozen in liquid nitrogen and either stored in −80°C or used directly. The frozen tissue samples were homogenized in 2 ml/mg tissue phosphate buffered saline pH 7.4 containing protease inhibitor cocktail (cOmplete, Roche) with the help of a tissue grinder. Cells in the homogenized tissue samples were lysed by adding 6× Laemmli buffer (TRIS-hydrochlorid 378 mM, 30% glycerol, 12% SDS and 0, 06% Bromphenolblue, 10% β-mercaptoethanol) to the samples and a 1–5 min incubation. Proteins were denaturated at 95°C for 5 min.

HEK293 cells were lysed in phosphate buffered saline pH 7.4 containing protease inhibitor cocktail (cOmplete, Roche) and 1% triton X-100. The lysis reaction was incubated 1 h at 4°C under rotation. After the lysis protein lysates were separated from cell debris by centrifugation at 15,000 rpm, 5 min at 4°C. 6× Laemmli buffer was added to the samples and proteins were denatured at 5 min at 95°C.

Protein homogenates were separated by SDS polyacryalmide gel electrophoresis (SDS PAGE) and blotted to nitrocellulose membrane overnight. Membranes were incubated 1.5 h in blocking solution of either 5% fish gelatin in PBS to avoid unspecific binding of antibodies and overnight at 4°C with a polyclonal antibody against RIM3γ (1:100; cite Alvarez-Baron et al., 2013) and a monoclonal antibody against β-tubulin (1:10,000; BD Pharmigen). Antibody staining was visualized by incubation with IRDye anti rabbit 680 nm IgG and IRDye 800-anti mouse IgG (LI-COR) in a dilution of 1:20,000 for 1 h and an infrared scanning system (Odyssey, Licor). Quantification of western blots was carried out using the analyze gels plugin of the FIJI software.

### Co-Immunoprecipitation

HEK293T cells were plated at a density of 1.5 × 10^5^ cells/dish and co-transfected (Ca^2+^-phosphate method) with the following plasmids: full-length untagged RIM2α and the HA-Ca_V_1.3 (aa 1509–2203), ZF-PDZ domain of RIM2α and HA-Ca_V_1.3 and C_2_A-C_2_B domain of RIM2α and HA-Ca_V_1.3. Forty-eight hours post-transfection cells were lysed for 1 h in ice cold lysis buffer (50 mM HEPES pH: 7.5, 150 mM NaCl, 1% Triton X-100) supplemented with proteinase inhibitors (Roche), followed by a short centrifugation step at 14.000 rpm/10 min/4°C. The clear supernatant was incubated for 2 h/4°C with HA-magnetic beads (Pierce) on a rotator. After the incubation time, beads were extensively washed with PBS-0.5% Triton X-100 buffer and boiled at 95°C/5 min in Laemmli buffer supplemented with β-ME. Proteins were resolved in SDS-PAGE gel (8%), followed by the protein transfer to the nitrocellulose membrane (Millipore). The detection of the proteins was performed using primary antibodies anti-mouse HA (Covance; 1:1000), anti-rabbit RIM1/2 (1:1000; provided by Frank Schmitz), followed by secondary antibodies IRDye 1:10,000 (goat anti-mouse 800 and goat anti-rabbit 680). The detection was achieved with an infrared imaging system (Odyssey, Li-cor).

### GST Pull-Down

The GST-fusion proteins (PDZ domain, C_2_A domain and C_2_B domain of RIM2α) were produced in *Escherichia coli* BL21-DE3 and purified using Glutathion-agarose beads (Sigma). The purification efficiency was assessed by Coomassie staining (Supplementary Figure S1). For the binding assay the HA-tagged C-terminal region of Ca_V_1.3α (aa 1509–2203) was overexpressed in HEK293T cells using either calcium-phosphate method or Lipofectamine2000 (Invitrogen). Forty-eight hours post transfection cells were lysed for 1 h in ice-cold lysis buffer (50 mM HEPES pH 7.4, 150 mM NaCl, 1% Triton X-100, Complete Protease Inhibitor Cocktail Tablets), centrifuged at 14,000 rpm/10 min/4°C and the resulting clear supernatant incubated for 2 h with GST and GST-fusion proteins. Beads were washed four times in PBS-0.5% Triton X-100 and proteins were eluted by boiling the beads in Laemmli buffer. Proteins were analyzed by WB using the Odyssey infrared imaging system.

### Patch-Clamp Recordings of Transiently Transfected HEK293/SK3-1 Cells

For electrophysiological recordings human embryonic kidney cells stably expressing the human small-conductance Ca^2+^-activated K^+^ channel (HEK293/SK3-1) were transfected at 30% confluence using the transfection reagent ExGen500 (Biomol) containing Ca_V_1.3_A2123V_α1 (Tan et al., 2011), β2a (GenBank accession number: NM053851), α2δ1 (GenBank accession number: NM012919), RIM2α (GenBank accession number: NM_001256383) and RIM3γ (GenBank accession number: NM_182929.2) according to the manufactures protocol. Thirty-six to sixty hours after transfection I_Ca_ were acquired at room temperature using an external solution containing the following (in mM): 150 CholineCl, 1 MgCl_2_, 10 HEPES, 10 CaCl_2_, 100 nM Apamin; pH 7.4 (adjusted with methanesulfonic acid), 300–310 mosmol. The internal solution contained the following (in mM): 140 N-Methyl-D-glucamine, 5 EGTA, 10 NaCl, 1 MgCl_2_, 10 HEPES, 2 MgATP; pH 7.4 (adjusted with NaOH), 290 mosmol. I_Ca_ was recorded using an EPC 10 Amplifier controlled by “Patchmaster” software (HEKA), low-pass filtered at 5 kHz, sampled at 50 kHz with R_Series_ of ≤ 10 MΩ after 70% compensation.

Conductance of Ca^2+^ channels was were derived from the I–V curves *G* = *I*/(*V*−*V*_rev_) (*V*_rev_ reversal potential of the Ca^2+^ current), was normalized to the maximal conductance (*G*_max_) and fitted to the following equation: *G*/*G*_max_ = 1/(1+*exp*^V_0.5_−V/k_act_^) to derive the potential of half maximal *I*_Ca_ activation (*V*_0.5_) and the activation slope factor of the Boltzmann function (*k*_act_).

### Immunofluorescence on Transiently Transfected HEK293/hSK3-1 Cells

For immunostaining of RIMs and Ca^2+^ channels in co-transfected HEK293/hSK3-1 cells, cells were fixed for 2 min at −20°C with 99% methanol. Primary antibodies were rabbit anti-Ca_V_1.3 (1:50, Alomone Labs), goat anti-RIM2 (1:200, sc-16677, Santa Cruz Biotechnology), mouse anti-RIM3 (1:100 (Alvarez-Baron et al., 2013)), which were detected by species-specific Abberior STAR 580 and 635 for STED images (all secondary antibodies: 1:200). Specimens were imaged using a Abberior Instruments laser-scanning confocal/STED microscope with a 1.4 NA, 100× oil-immersion (STED) objective using excitation wavelengths of 561 and 640 nm. For STED microscopy a STED laser of 775 nm up to 1.2 W was used at a pulse rate of 40 MHz achieving a resolution of <30 nm. Every staining was repeated at least three times and representative images are shown.

### Patch-Clamp Recordings of IHCs

These recordings were performed in the apical coil of the organ of Corti isolated from mice at the age of P15–P20 using the perforated-patch configuration at room temperature. The following solutions were used: extracellular solution (in mM): 113 NaCl, 2.8 KCl, 35 TEA-Cl, 1 CsCl, 1 MgCl_2_, 2 CaCl_2_, 10 NaOH-HEPES, 11.3 D-glucose at pH 7.3; intracellular solution (in mM): 135 Cs-gluconate, 10 TEA-Cl, 10 4-aminopyridine, 1 MgCl_2_, 10 CsOH-HEPES and 300 μg/ml amphotericin. Traces were low-pass filtered at 2.9 kHz recorded at a sampling rate of 50 kHz, underwent offline liquid junction potential correction and for being accepted required a R_Series_ <30 MΩ for analysis. Capacitance recordings were performed as previously published (Moser and Beutner, 2000).

### Auditory Brainstem Recordings

For recordings of Auditory Brainstem Recordings (ABRs), mice were anesthetized with a combination of i.p.-administered ketamine (125 mg/kg) and xylazine (2.5 mg/kg). The core temperature was maintained constant at 37°C using a heat blanket (Hugo Sachs Elektronik–Harvard Apparatus). For stimulus generation, presentation and data acquisition, we used the TDT II System run by BioSig software (Tucker Davis Technologies, MathWorks). Tone bursts (4/6/8/12/16/24/32 kHz, 10-ms plateau, 1-ms cos2 rise/fall) or clicks of 0.03 ms were presented at 40 Hz (tone bursts) or 20 Hz (clicks) in the free field ipsilaterally using a JBL 2402 speaker. The difference potential between vertex and mastoid subdermal needles was amplified 50,000-fold, filtered (400–4000 Hz), and sampled at a rate of 50 kHz for 20 ms for a total of 1300 times to obtain two mean ABR traces for each sound intensity. Hearing threshold was determined with 10-dB precision as the lowest stimulus intensity that evoked a reproducible response waveform in both traces by visual inspection by two independent observers.

### Statistical Analysis

Data are presented as mean ± SEM. For statistical comparisons Student’s *t*-test was used to compare normally distributed samples with indistinguishable variance or alternatively Wilcoxon rank-sum test was used as non-parametric test. For multiple comparisons of normally distributed data (assessed by Kolmogorov-Smirnov test) one-way ANOVA with *post hoc* Holm-Šídák were performed; *p* ≤ 0.05 was accepted as statistically significant and is indicated by * *p* < 0.01 by ** and *p* < 0.005 by ***.

## Results

### Biochemical Evidence for a Direct Interaction of RIM2α and RIM3γ with Ca_V_1.3α

**Figure 1:**
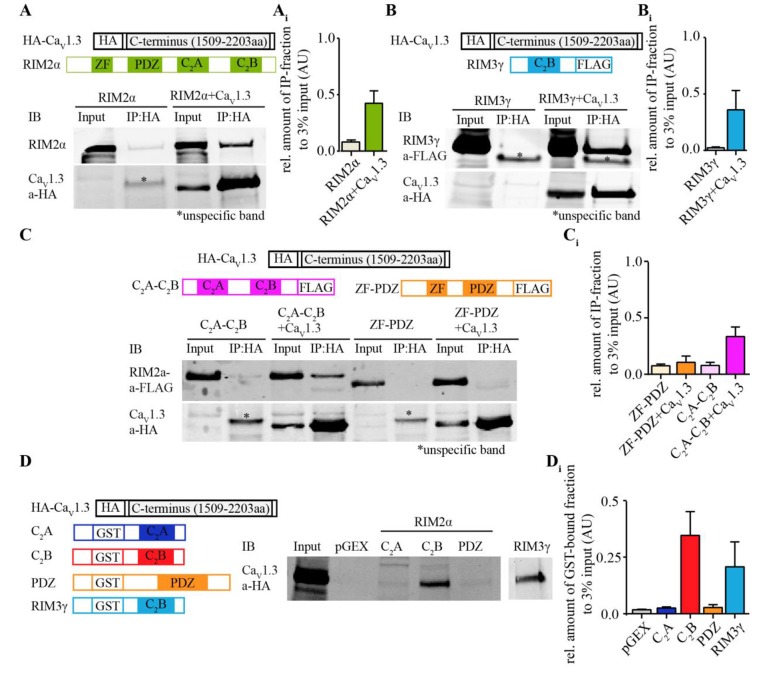
Rab interacting molecules 2α (RIM2α) interacts with Ca_V_1.3 via C_2_-domain binding to the Ca_V_1.3α C-terminus. **(A)** Schematic representation of RIM2α and HA-tagged Ca_V_1.3 C-terminus (top). Immunoblot (IB) of an exemplary co-immunoprecipitation assay from co-transfected HEK293T cell lysates shows that full length RIM2α co-immunoprecipitated with the C-terminal region of Ca_V_1.3 (bottom, input 3%). **(A_i_**) Quantifications of co-immunoprecipitated RIM2α with the HA-tagged C-terminal region of Ca_V_1.3 (*N* = 3). **(B)** Schematic representation of RIM3γ and HA-tagged Ca_V_1.3 C-terminus (top). IB of an exemplary co-immunoprecipitation assay from co-transfected HEK293T cell lysates, showing that the C_2_B domain of RIM3γ suffices to co-immunoprecipitate with the C-terminal region of Ca_V_1.3 (bottom, input 3%). **(B_i_**) Quantifications of co-immunoprecipitated RIM3γ with the HA-tagged C-terminal region of Ca_V_1.3 (*N* = 2). **(C)** Schematic representation of fusion proteins of RIM2α subdomains, RIM3γ and Ca_V_1.3 C-terminus as used for the binding assays (top). Immunoblot (IB) of an exemplary co-immunoprecipitation assay from co-transfected HEK293T cell lysates, showing that the peptide containing the RIM2α C_2_-domains, but not the RIM2α ZF-PDZ peptide co-immunoprecipitated with HA-tagged C-terminal region of Ca_V_1.3α (bottom, input 3%). **(C_i_**) Quantifications of co-immunoprecipitated N-(ZF-PDZ, *N* = 3) or C-terminal (C_2_A-C_2_B, *N* = 3) domains of RIM2α and RIM3γ with the C-terminal region of Ca_V_1.3. **(D)** Schematic representation of fusion proteins used for the GST pull-down assay (Left). IB of an exemplary GST pull-down assay of HA-tagged Ca_V_1.3 (1509–2203aa) overexpressing HEK293T cell lysates, showing that the C_2_B-domain of RIM2α (GST-RIM2α C_2_B), but not the C_2_A or PDZ domain of RIM2α (GST-RIM2α C_2_A and GST-RIM2α PDZ) pulled down Ca_V_1.3 and were detected by an anti-HA antibody (right, input 3%). **(D_i_**) Quantification of GST-bound fraction of HA-tagged Ca_V_1.3 pulled down by respective RIM2α and RIM3γ domains (*N* = 4). Note that the RIM2α-C_2_B and RIM3γ pulled down Ca_V_1.3 while the RIM2α-C_2_A and -PDZ domains did not.

We tested for a direct interaction of Ca_V_1.3α and RIM2α by co-immunoprecipitation from transfected HEK293T cells and by GST-pull down assays (Figure 1, Supplementary Tables S1, S2). We found that full-length RIM2α was co-immunoprecipitated with an HA-tagged version of the C-terminus of Ca_V_1.3α1 (Figure 1A). However, unlike for Ca_V_2.1α1 and Ca_V_2.2α1 (Kaeser et al., 2011), a construct containing the RIM2α-PDZ domain (here also including the ZF domain) did not bind the Ca_V_1.3α-C-terminus (Figure 1C). Instead, the C-terminus of RIM2α, containing two C_2_ domains, C_2_A and C_2_B, co-immunoprecipitated with the Ca_V_1.3α-C-terminus (Figure 1C). In order to further narrow down the site of interaction of RIM2 we performed GST-pulldown assays. Only the GST-tagged RIM2α-C_2_B domain but not the RIM2α-C_2_A and—PDZ domains bound to the HA-tagged Ca_V_1.3α1-C-terminus (Figure 1D). Similar findings were obtained for RIM3γ (Figure 1B) indicating that this interaction of the Ca_V_1.3α1-C-terminus generalizes to C_2_B domains of other RIMs.

### Co-Expression of RIM2α or RIM3γ Increases the Current Density Mediated by “IHC-Like” Ca_V_1.3 Channel Complexes in HEK293/SK3-1 Cells

**Figure 2:**
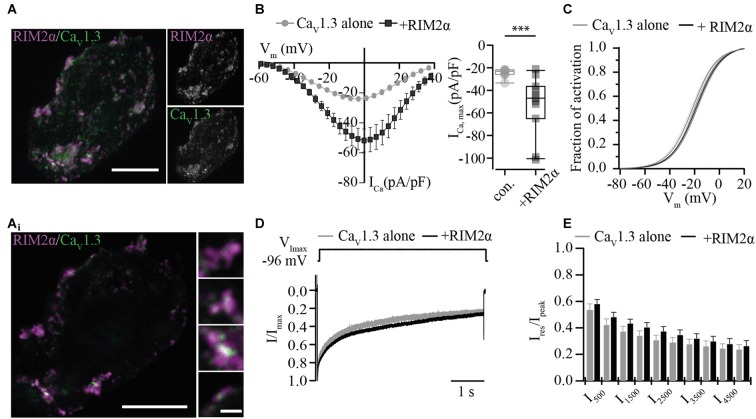
RIM2α positively regulates functional expression of Ca_V_1.3 in HEK 293/SK3-1 cells. **(A)** Confocal maximum projection of a representative HEK293/SK3-1 cell co-transfected with Ca_V_1.3 (green) and RIM2α (magenta; Scale bar: 10 μm). **(A_i_**) 2D STED image of **(A)** showing overlap RIM2α and Ca_V_1.3 immunofluorescence indicative of co-localization (Identical scale in all enlarged images; Scale bar: 1 μm). **(B)** The co-expression of RIM2α increases the Ca^2+^-current density amplitude in transiently transfected HEK293/SK3-1 cells: average I-V traces, depicted as current densities of HEK293/SK3-1 transfected with either Ca_V_1.3 alone (con. for control, gray, *n* = 9) or cells co-transfected with RIM2α (black, *n* = 11; left). Summary plot of maximum current densities shown as box plot (10, 25, 50, 75 and 90% percentiles) overlaid with individual data points (right). Note the two-fold increase in maximum current-density amplitude in the presence of RIM2α (***p < 0.005, Wilcoxon rank-sum test). **(C)** Voltage-dependence of activation curve derived from **(B)** in the presence or absence of RIM2α. The voltage-dependence of activation curve is mildly shifted towards more positive potentials in presence of RIM2α ( *p* < 0.05, Student’s *t*-test). **(D,E)** Ca^2+^-current inactivation is not affected by co-expression of RIM2α: average I_Ca_ traces recorded in the presence (*n* = 11) or absence (*n* = 9) of RIM2α after step depolarization to the voltage of maximal Ca^2+^-currents ( V_Imax_) for 5 s. For a better comparison traces were normalized to the maximum current (I_peak_). Residual Ca^2+^-currents (I_res_) were indistinguishable between recording conditions ( *p* > 0.05, One-way ANOVA with *post hoc* Holm-Sidak correction).

In order to assess the functional relevance of the direct interaction of RIM isoforms with the Ca_V_1.3α1 C-terminus, we studied the effect of full-length RIM2α or RIM3γ on voltage-gated Ca^2+^-currents mediated by “IHC-like” Ca_V_1.3 channels (Ca_V_1.3α1, Ca_V_β2a and Ca_V_α2δ1) in HEK293 cells. We employed HEK293 cells that stably express the Ca^2+^-activated small-conductance K^+^ channel (SK3-1) as a negative feedback in an attempt to improve the viability of the cells by limiting toxic Ca^2+^-influx. We chose to work with the rat Ca_V_1.3α clone used by Tan et al. (2011) (corrected for a C-terminal mutation) and the Ca_V_β2a in order to mimic the IHC Ca^2+^ channel complex (Platzer et al., 2000; Brandt et al., 2003; Neef et al., 2009) as closely as possible. Moreover, use of the Ca_V_β2a is expected to minimize possible effects of the RIM2-Ca_V_β interaction on channel gating (Gebhart et al., 2010). Immunofluorescence analysis of Ca_V_1.3 and RIM2 expression in HEK293/SK3-1 cells showed partial overlap of signals at or near the plasma membrane (Figure 2A), indicative of a co-localization of both proteins and compatible with their interaction.

For the electrophysiological analysis we only included recordings with current densities, the Ca^2+^-current normalized to the cell capacitance, exceeding 20 pA/pF in order to increase the signal-to-noise ratio. Under these conditions the current density was nearly doubled when co-expressing RIM2α (Figure 2B), suggesting a positive regulation of Ca_V_1.3 channel plasma membrane expression. The voltage-dependence of Ca_V_1.3 channel activation was shifted toward more depolarized potentials by 3 mV (Figure 2C, Supplementary Table S2), while the inactivation of the Ca^2+^-current was neither significantly changed for its early nor its later components (Figures 2D,E).

**Figure 3:**
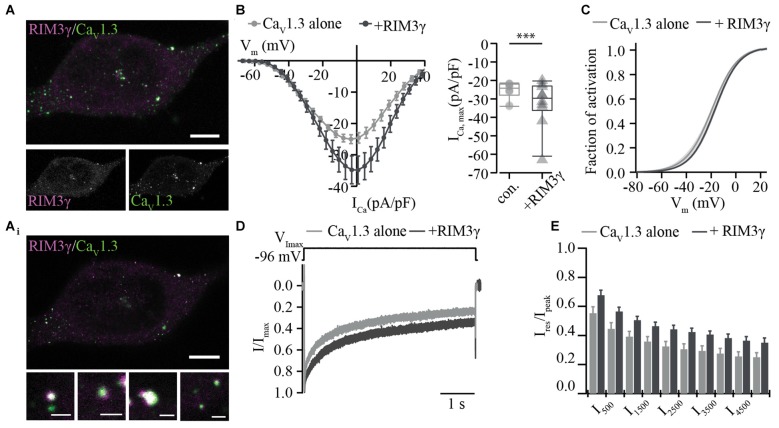
RIM3γ positively regulates functional expression of Ca_V_1.3 in HEK 293/SK3-1 cells. **(A)** Confocal maximum projection of a representative HEK293/SK3-1 cell co-transfected with Ca_V_1.3 (green) and RIM*3γ* (magenta; Scale bar: 5 μm). **(A_i_**) 2D STED image of **(A)** showing overlap of RIM3γ and Ca_V_1.3 immunofluorescence indicative of co-localization (Scale bar of enlarged images: 0.5 μm). **(B)** Elevated Ca^2+^-current amplitudes in the presence of RIM3γ in transfected HEK293/SK3-1 cells: average I-V traces recorded in HEK293/SK3-1 in the presence (dark gray, *n* = 10) or absence (con. for control, gray, *n* = 10) of RIM3γ (left). Summary plot of maximum current densities shown as box plot (10, 25, 50, 75 and 90% percentiles) overlaid with individual data points (right). Note the increase in maximum current density amplitude in the presence of RIM3γ (***p < 0.005, Wilcoxon rank-sum test). **(C)** Voltage-dependence of activation derived from B is not shifted in the presence of RIM3γ ( *p* > 0.05, Student’s *t*-test). **(D,E)** Ca^2+^-current inactivation is not affected by co-expression of RIM3γ: average Ca^2+^-current traces recorded in the presence (*n* = 8) or absence (*n* = 10) of RIM3γ. Residual Ca^2+^-currents inactivating were indistinguishable between recording conditions ( *p* > 0.05; One-way ANOVA with *post hoc* Holm-Sidak correction).

We then tested whether the RIM3γ that only contains the C_2_B domain also promotes membrane expression of Ca_V_1.3 channels. Immunofluorescence analysis of Ca_V_1.3 and RIM3γ in HEK293/SK3-1 showed partial overlap of signals at or near the plasma membrane (Figure 3A), indicative of a co-localization of both proteins and compatible with their interaction. We found a mild but significant increase in maximum Ca^2+^-current densities in HEK293/SK3-1 cells co-expressing RIM3γ (Figure 3B). The voltage-dependence of Ca_V_1.3 channel activation and Ca^2+^-current inactivation remained unchanged (Figures 3C–E, Supplementary Table S2). In summary, both RIM2α and RIM3γ that are present at IHC AZs increase Ca^2+^-current densities in HEK293/SK3-1 cells expressing an IHC-like Ca^2+^ channel complex. Since we used the palmitoylated Ca_V_β2a subunit, for which previous work investigating the interaction of RIM and Ca_V_β subunits found the least effect, we speculate that this increase reflects a positive regulation of membrane expression via the direct interaction between the C_2_B domain of RIM2α and RIM3γ and the C-terminus of Ca_V_1.3α1. As both RIM isoforms, RIM2α and RIM3γ, are present at IHC AZs, these interactions might be functionally relevant in IHCs.

### Does RIM3 have a Functional Role at IHC AZs?

**Figure 4:**
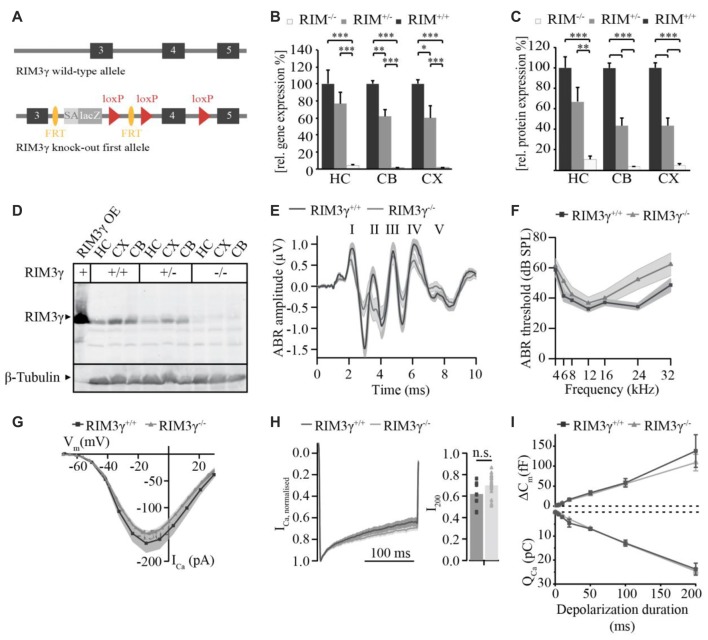
Analysis of RIM3γ knock-out mice shows that RIM3γ is not required for the function of inner hair cell (IHC) ribbon synapses. **(A)** Cartoon depicting the RIM3γ wild-type and targeted allele, in which a gene trap cassette was inserted in the intronic region between exon 3 and 4. The gene trap cassette consists of a promoterless En2 splice acceptor site followed by an IRES-lacZ gene cassette. Splicing of this gene trap cassette to the end of RIM3γ exon 3 results in disruption of RIM3γ expression. **(B)** Quantitative real time RT-PCR of wild-type, heterozygous and homozygous *RIM3*γ^−/−^ mice revealed reduced gene transcript levels in mRNA prepared from hippocampus (HC), cerebellum (CB) and cortex (CX; *N* = 10 animals per group). **(C)** Quantification immunoblot (*N* = 5 animals per group). Significance: two-way ANOVA, Bonferoni *post hoc* Test *p < 0.05, **p < 0.01 and ***p < 0.001. **(D)** Immunoblot of *RIM3*γ^−/−^ mice, showed a pronounced reduction in protein levels in HC, CB and CX. To verify RIM3γ reactivity of the antibody and the identity of the band analyzed, HEK293 cell lysates overexpressing the RIM3γ were included in the analysis (RIM3γOE). **(E)** Grand average of auditory brainstem responses (ABR) of *RIM3*γ^−/−^ (*N* = 9) and littermate control (*N* = 7) animals show normal wave I amplitudes and latencies in the absence of RIM3γ. The shaded area represents the SEM and roman letters indicate the five characteristic ABR waves representing auditory nuclei along the auditory pathway. **(F)** Mean ABR hearing thresholds of *RIM3*γ^−/−^ and control animals show near normal hearing thresholds over the whole frequency range in the absence of RIM3γ. Shaded areas represent the SEM. **(G)** Mean current-voltage relationship of Ca^2+^-currents in *RIM3*γ^−/−^ (*n* = 20) and littermate control (*n* = 9) IHCs. Shaded areas represent the SEM. Note that the maximum current amplitude is comparable between genotypes ( *p* > 0.05, Student’s *t*-test). **(H)** Mean Ca^2+^-current trace of *RIM3*γ^−/−^ (*n* = 24) and control (*n* = 14) IHCs (left) showing comparable inactivation kinetics summarized in single value plot for the residual Ca^2+^-current after 200 ms (right, *p* < 0.05, Student’s *t*-test). **(I)** Mean ± SEM. cell capacitance increments (∆C_m_) with respective Ca^2+^-charge (Q_Ca_) upon depolarizations of increasing durations of *RIM3*γ^−/−^ (*n* = 24) and control (*n* = 14) IHCs. Both ∆C_m_ and Q_Ca_ are unchanged in the absence of RIM3γ.

In previous work we showed, that RIM2α, RIM2ß and RIM3γ but not RIM1 are expressed in IHCs and localize at the ribbon synapse (Jung et al., 2015). In order to investigate the role of RIM3γ in IHC synaptic transmission we generated and analyzed constitutive RIM3γ knock-out mice (*RIM3*γ^−/−^). *RIM3*γ^−/−^ mice were generated by targeting ES cells with a gene trap cassette, in which insertion of a splice acceptor-lacZ gene trap disrupts the endogenous RIM3γ transcripts resulting in a constitutive knock-out (Figure 4A). In order to verify that the insertion of the splice acceptor-cassette indeed abolishes the expression of functional RIM3γ, we characterized transcripts and protein expression levels in brains of wild-type, heterozygous and homozygous *RIM3*γ^−/−^ mice. The level of transcripts was assessed by quantitative real time RT-PCR of RIM3γ mRNA prepared from hippocampus (HC), cerebellum (CB) and cortex (CX). RIM3γ transcript levels were reduced to about 60% in heterozygous and almost completely abolished in homozygous *RIM3*γ^−/−^ mice in all brain areas (Figure 4B). To analyze if these reduced transcript levels result in the ablation of the protein quantitative immunoblotting of homogenates from hippocampus, cerebellum and cortex were performed. Stainings of the immunoblots with a RIM3γ-specific antibody revealed, that the gene trap had successfully abolished RIM3γ protein expression (Figures 4C,D). Hearing was tested via auditory brainstem responses (ABR) by presenting acoustic stimuli and recording synchronized neuronal activity. Here, the individual ABR waves, indicated by roman letters, reflect the processing at various stages of the early auditory pathway (Figure 4E). Amplitude and latency of ABR wave I, which represents the synchronized firing activity of spiral ganglion neurons (compound action potential of the spiral ganglion), was unaffected by the disruption of RIM3γ. In addition, ABR thresholds were comparable between RIM3γ^−/−^ and littermate controls (Figure 4F), suggesting a minor if any role of RIM3γ in sound encoding.

We note that a mild hearing impairment was found in RIM2 knock-out mice, while Ca^2+^-current amplitudes as well as sustained exocytosis were reduced by 50% (Jung et al., 2015). Therefore, we recorded Ca^2+^-currents and membrane capacitance increments upon depolarizations in perforated-patch configuration. However, we only found non-significant trends towards reduced Ca^2+^-current amplitudes (Figure 4G) and a tendency towards enhanced Ca^2+^-current inactivation (measured as ratio of the residual Ca^2+^-current after 200 ms depolarizations and the initial current, i.e., peak-normalized I_200_, Figure 4H) in RIM3γ-deficient IHCs. Moreover, we probed exocytic changes of membrane capacitance (∆C_m_) in response to depolarizations of varying durations and found indistinguishable fast (<20 ms depolarizations) and sustained exocytosis between genotypes (Figure 4I). In summary, while RIM3γ promotes Ca_V_1.3 membrane expression in HEK293 cells and is expressed at IHC AZs, it seems largely dispensable for IHC presynaptic function.

## Discussion

RIM proteins are multifunctional proteins that positively regulate vesicle tethering and Ca^2+^ channel clustering at AZs. Here, we studied whether RIM2α and RIM3γ, both expressed at IHC AZs, directly interact with the pore-forming Ca_V_1.3α Ca^2+^ channel subunit that mediates stimulus-secretion coupling at IHC synapses. Based on co-immunoprecipitation, GST-pull-down assays, fluorescence microscopy of protein co-localization and electrophysiology in HEK293 cells, we indicate that RIM2α and RIM3γ directly bind to the C-terminus of the pore-forming Ca_V_1.3α1 subunit most likely via their C_2_B domain. Both, RIM2α and RIM3γ, enhance the Ca_V_1.3 Ca^2+^-current when co-expressed in HEK293/SK3-1 cells. While, RIM2α is required for establishing a large complement of Ca_V_1.3 Ca^2+^ channels at IHC AZs, the presence of RIM3γ seems to be dispensable for Ca^2+^-influx and exocytosis in IHCs.

### Interaction of RIMs and Ca_V_1.3 Ca^2+^ Channels in HEK293 cells

A RIM-mediated up-regulation of Ca^2+^ channel density at AZ was reported in hair cells, hippocampal neurons and the calyx of Held and can be attributed to various modes of direct and indirect interaction between RIMs and Ca^2+^ channels. To date, two direct interaction sites of RIMs and specific Ca^2+^ channel isoforms were reported. RIMs were proposed to exhibit a PDZ-domain dependent interaction with the pore-forming Ca_V_α1 subunit of Ca_V_2.2 and Ca_V_2.1 channels (Ca_V_2.X, Kaeser et al., 2011) and bind to the synprint motif of Ca_V_2.2 and Ca_V_1.2 channels via the C-terminal C_2_A and C_2_B domains (Coppola et al., 2001). However, neither of the described mechanism seems to apply to Ca_V_1.3 channels (Coppola et al., 2001; Kaeser et al., 2011), the predominant Ca^2+^ channel isoforms at IHC ribbon synapses, that similar to the calyx of Held (Han et al., 2015) display a substantial Ca^2+^-current reduction in the absence of RIM2 (Jung et al., 2015). For Ca_V_1.3, as well as for Ca_V_1.2, Ca_V_2.1 and Ca_V_2.2 channels, a C-terminal C_2_-domain dependent interaction of RIM with the auxiliary Ca_V_β subunit was shown to regulate the biophysical properties of Ca^2+^ channels in heterologous expression systems (Kiyonaka et al., 2007; Gebhart et al., 2010; Gandini et al., 2011). In addition, RIMs are indirectly linked to Ca^2+^ channels by RIM-binding proteins, which seem to be dispensable for the regulation of membrane expression of Ca^2+^ channels in central synapses (Hibino et al., 2002; Kaeser et al., 2011; Liu et al., 2011; Acuna et al., 2015). The findings of the present study support a direct interaction of the C_2_B domain of RIM2α and RIM3γ with the C-terminus of the Ca_V_1.3α-subunit. In keeping with the notion of Kaeser et al. (2011), we did not observe binding of the RIM2 PDZ-domain to the ITTL-site of Ca_V_1.3α, that also diverges from the consensus-motif for RIM1/2 PDZ-domains (Kaeser et al., 2011; DDWC (Ca_V_2.1); DHWC (Ca_V_2.2); DDKC (Ca_V_2.3) vs. ITTL (Ca_V_1.3)). This is interesting in the light of the established interaction of the Ca_V_1.3α-subunit with other PDZ-domain proteins (Calin-Jageman et al., 2007; Gregory et al., 2011). The C_2_B-domain of all RIMs contains a short Lysine-rich amino acid sequence that is also found in Synaptotagmin 1 (Perin et al., 1990; Coppola et al., 2001; Wang and Südhof, 2003) and Munc13-1 (Calloway et al., 2015), which interacts with the synprint site of Ca_V_2 channels. However, the synprint site characterized in Ca_V_1.2 (Wiser et al., 1999), P/Q- (Catterall, 1999) and N-type (Sheng et al., 1997) Ca^2+^ channels seems to be lacking in Ca_V_1.3α1 (Coppola et al., 2001). Therefore, our results indicate the presence of a novel RIM binding motif in the C-terminus of Ca_V_1.3α, which will have to be mapped in further studies.

Our analysis of biophysical Ca_V_1.3α properties in HEK293 cells took advantage of the constitutive presence of a negative feedback to Ca^2+^-influx by the small conductance Ca^2+^-activated K^+^ channel SK3-1, which increased the yield of Ca_V_1.3α-positive cells that were in good condition. This raised our confidence in interpreting an increase in current density as the enhanced membrane expression of Ca_V_1.3α when co-expressed with RIM2α or RIM3γ. We chose to compare current densities above a threshold of 20 pA/pF for signal to noise considerations, but note that current densities were typically larger for RIM co-expressing cells also below this margin.

A RIM1 mediated upregulation of Ca^2+^ channel densities in co-expression studies in heterologous expression systems was previously reported for Ca_V_2.1 and Ca_V_2.2 channels (Kiyonaka et al., 2007). However for Ca_V_1.3, an enhanced Ca^2+^-current density was either not detected in the presence of RIM1 (Gandini et al., 2011) or not reported in the presence of RIM2 (Gebhart et al., 2010). The discrepancy between this and the previous study by Gandini et al. (2011) might result from deviating RIM proteins (RIM1 vs. RIM2α, RIM3γ) and Ca_V_1.3α1 isoforms used for these experiments (corrected vs. uncorrected Ca_V_1.3 rat clone) or the differing IV protocols applied (steady-state IV after 30 ms here vs. 2 s). Here, we favor the interpretation that the increased current density resulted from enhanced plasma membrane expression due to direct RIM-C_2_B interaction with the C-terminus of Ca_V_1.3α but cannot rule out an additional effect of RIM via Ca_V_ß-dependent positive regulation of Ca_V_1.3α (Gebhart et al., 2010). While enhanced current density could in principle also reflect an increase in open probability by RIM-Ca_V_1.3α interaction, the depolarized shift of Ca_V_1.3α activation seems to argue against this. Instead, such a shift likely indicates a negative regulation of Ca_V_1.3α gating by RIM interaction potentially by impacting on the function of the Ca_V_1.3α C-terminus (Bock et al., 2011). In summary, experiments on heterologously co-expressed Ca_V_1.3α and RIMs indicate a functionally relevant interaction involving the C_2_B domain of RIM and the C-terminus of Ca_V_1.3α. Further studies will need to establish the precise molecular mechanism and affinity of this interaction.

### Role of RIMs in Promoting Synaptic Ca^2+^-Influx in IHCs

The increased Ca_V_1.3 Ca^2+^-current density in HEK293/SK3-1 cells upon co-expression of RIM2α and RIM3γ is consistent with the notion that RIMs are positive regulators of plasma membrane expression of Ca^2+^ channels as proposed based on genetic disruption of RIM function for several presynaptic terminals (Han et al., 2011, 2015; Kaeser et al., 2011; Kintscher et al., 2013; Jung et al., 2015). In IHCs, genetic deletion of all RIM2 isoforms caused a robust reduction of IHC Ca^2+^-influx (by approximately 50%), while the selective disruption of RIM2α diminished IHC Ca^2+^-influx by only 17% (Jung et al., 2015). This indicated that RIM2ß and/or RIM2γ promote the clustering of Ca^2+^ channels at IHC AZ in an additive manner with RIM2α potentially facilitated by the formation of RIM dimers (Guan et al., 2007). Our present findings of an interaction of the RIM C_2_B domain with the C-terminus of Ca_V_1.3α and a positive regulation of Ca_V_1.3 Ca^2+^-current density by RIM3γ in HEK293 cells suggests a putative presynaptic function, even though the protein is present pre- and postsynaptically (Liang et al., 2007; Alvarez-Baron et al., 2013). Indeed, we found expression of RIM3γ in IHCs at the mRNA and protein levels (Jung et al., 2015). However, genetic deletion of RIM3γ left IHC Ca^2+^-influx and hearing unaffected. The lack of a significant sound coding phenotype in the RIM3γ knock-out mice might be related to a predominant role of the long RIM2 isoforms that co-exist with RIM3γ at the IHC AZ. We speculate that their interaction with the various CAZ proteins poises them to critically determine the number of AZ tethered Ca^2+^ channels, while the short RIM3γ exerts more auxiliary function. A compensatory scenario was previously observed at the Calyx of Held synapse, where RIM1 and RIM2 possess the ability to largely replace each other (Han et al., 2015). Further studies investigating RIM2/RIM3 double-knock-out mice will be required to elucidate a potential contribution of RIM3γ in Ca_V_1.3 clustering at IHC AZs.

## Author Contributions

MMP, SS and TM designed the study. MMP performed electrophysiological recordings, immunohistochemistry and STED microscopy of heterologously expressed Ca^2+^ channels and IHC electrophysiology. A-MO performed *in vitro* interaction studies and immunohistochemistry of HEK cells. SJ performed IHC electrophysiology. KM generated the RIM3 KO mice. MMP, A-MO, KM and SJ analyzed the data. MMP, A-MO, SJ, SS and TM prepared the manuscript.

## Conflict of Interest Statement

The authors declare that the research was conducted in the absence of any commercial or financial relationships that could be construed as a potential conflict of interest. The reviewer MP and handling Editor declared their shared affiliation, and the handling Editor states that the process nevertheless met the standards of a fair and objective review.

## Supplementary Material

FIGURE S1 Exemplary SDS polyacryalmide gel electrophoresis (SDS-PAGEs) after Coomassie staining. **(A)** Visualized GST-bound protein fraction obtained by GST-pulldown assays. Protein bands of the respective rab interacting molecules 2 (RIM2) domains are indicated by the blue arrow heads. Protein amount loaded is indicated below. **(B)** GST-bound protein fraction of RIM3γ visualized in exemplary SDS-PAGE after Coomassie staining. The RIM3γ specific protein band is indicated by the blue arrow.
