# Supplementary material for: Rab Interacting Molecules 2 and 3 Directly Interact with the Pore-Forming CaV1.3 Ca2+ Channel Subunit and Promote Its Membrane Expression
Source: Front Cell Neurosci. 2017 Jun 8;11:160. doi: 10.3389/fncel.2017.00160 (PMC5462952; doi:10.3389/fncel.2017.00160)
Supplement: Supplementary file 1 [file Table_1.docx]

| Plasmid name | Backbone | Fragment | Primers (5’-3’) | Restr. enzyme |
| --- | --- | --- | --- | --- |
| CMV-ZF-PDZ-FLAG | CMV-MCS  (FLAG-C terminus) | ZF-PDZ  (1-719) |  |  |
| CMV-C2A-C2B-FLAG | CMV-MCS  (FLAG-C terminus) | C2A-C2B  (708-1555) | Fw:GCGtctagaACCATGTCTATATCGGTTACCTC  Rv: GCGgtcgacTGAACGAGAGTAAGAAGGTCC | XbaI  SalI |
| CMV-HA-Cav1.3 | CMV-MCV  (HA-N terminus) | Cav1.3  (1509-2203) | Fw: GCGtctagaGACAATTTTGACTATCTGACGAGG  Rv: GCGgtcgacTCACAAGGTGGTGATGCAAATCAT  TTC | XbaI  SalI |

**Table S1.** List of subcloned plasmids used for the biochemical binding assays including vectors backbones, fragment regions and cloning primers. RIM domains were subcloned from RIM2α (Rattus norvegicus, CMV-RIM2α); C-terminus of the long isoform of CaV1.3 was used for biochemical binding experiments (Rattus norvegicus, Tan et al., 2011, corrected for C-terminal mutation).
