# Supplementary material for: Rab Interacting Molecules 2 and 3 Directly Interact with the Pore-Forming CaV1.3 Ca2+ Channel Subunit and Promote Its Membrane Expression
Source: Front Cell Neurosci. 2017 Jun 8;11:160. doi: 10.3389/fncel.2017.00160 (PMC5462952; doi:10.3389/fncel.2017.00160)
Supplement: Supplementary file 2 [file Table_2.docx]

| Constructs | Current density (pA/pF) | p-value | V_h_ (mV) | p-value | k_act_ (mV) | p-value |
| --- | --- | --- | --- | --- | --- | --- |
| Ca_V_1.3α1/Ca_V_β2a/α2δ | - 24.91 ± 1.38 | 0.0032 WRST | - 21.61 ± 1.14 | 0.049 STT | 9.32 ± 0.36 | 0.55 STT |
| Ca_V_1.3 + RIM2α | - 52.90 ± 7.93 |  | - 18.72 ± 1.06 |  | 9.67 ± 0.19 |  |
| Ca_V_1.3α1/Ca_V_β2a/α2δ | - 25.66 ± 1.44 | 0.0236 WRST | - 19.54 ± 1.04 | 0.075 STT | 10.18 ± 0.40 | 0.29 STT |
| Ca_V_1.3 + RIM3γ | - 35.25 ± 4.47 |  | - 16.60 ± 1.14 |  | 9.69 ± 0.26 |  |

**Table S2.** Summary of current amplitudes and biophysical properties of Ca_V_1.3 in presence or absence of RIMs. Values are presented as mean ± SEM. Wilcoxon rank sum test is abbreviated as WRST and Student’s t-test as STT.
