# Supplementary figures and images for: Rab Interacting Molecules 2 and 3 Directly Interact with the Pore-Forming CaV1.3 Ca2+ Channel Subunit and Promote Its Membrane Expression
Source: Front Cell Neurosci. 2017 Jun 8;11:160. doi: 10.3389/fncel.2017.00160 (PMC5462952; doi:10.3389/fncel.2017.00160)

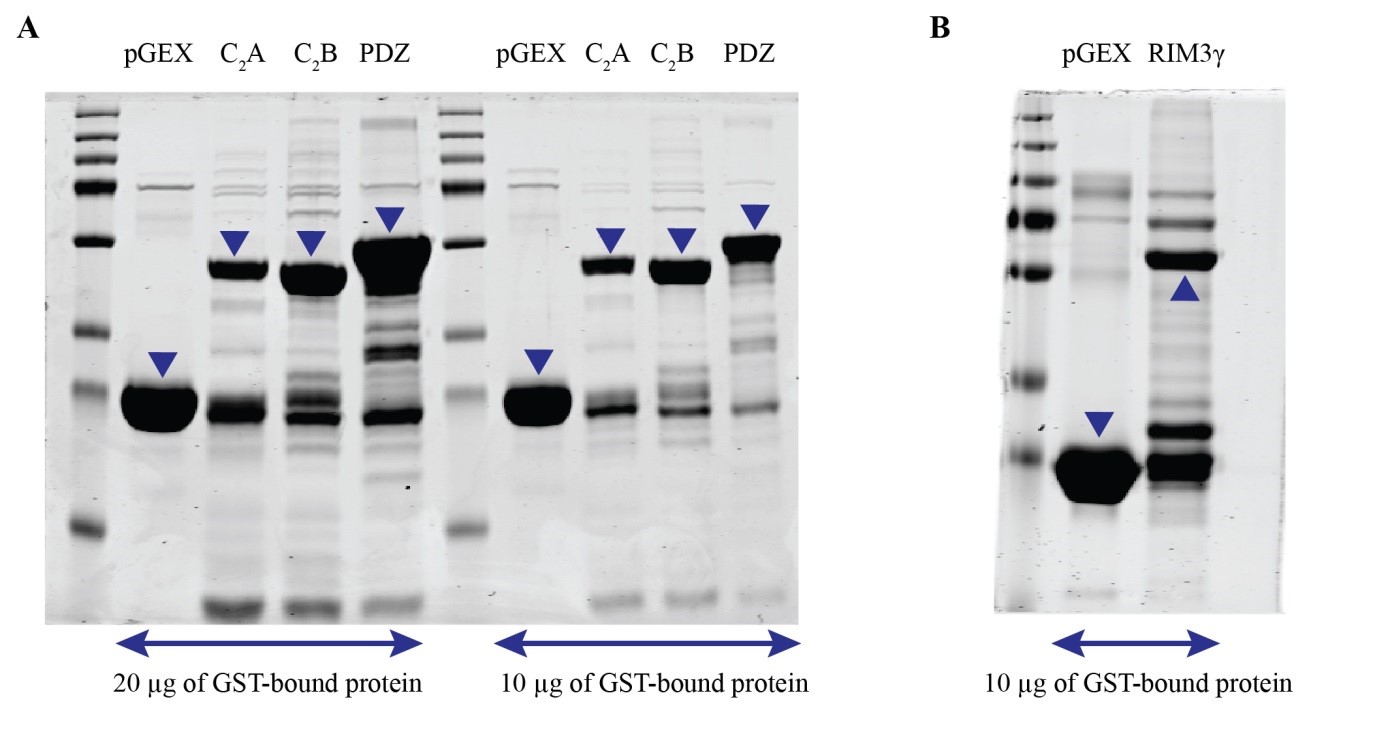

Supplement: FIGURE S1 — Exemplary SDS polyacryalmide gel electrophoresis (SDS-PAGEs) after Coomassie staining. (A) Visualized GST-bound protein fraction obtained by GST-pulldown assays. Protein bands of the respective rab interacting molecules 2 (RIM2) domains are indicated by the blue arrow heads. Protein amount loaded is indicated below. (B) GST-bound protein fraction of RIM3γ visualized in exemplary SDS-PAGE after Coomassie staining. The RIM3γ specific protein band is indicated by the blue arrow. [file Image_1.jpeg]
